# Honey, Gellan Gum, and Hyaluronic Acid as Therapeutic Approaches for Skin Regeneration

**DOI:** 10.3390/biomedicines13020508

**Published:** 2025-02-18

**Authors:** Patrícia Sousa, Alicia Moreira, Bruna Lopes, Ana Catarina Sousa, André Coelho, Alexandra Rêma, Maria Balça, Luís Atayde, Carla Mendonça, Lucília P. da Silva, Cristiana Costa, Alexandra P. Marques, Irina Amorim, Rui Alvites, Filipa Batista, Filipa Mata, João Transmontano, Ana Colette Maurício

**Affiliations:** 1 Departamento de Clínicas Veterinárias, Instituto de Ciências Biomédicas de Abel Salazar (ICBAS), Universidade do Porto (UP), Rua de Jorge Viterbo Ferreira, n° 228, 4050-313 Porto, Portugal; pfrfs_10@hotmail.com (P.S.); alicia.moreira.1998@gmail.com (A.M.); brunisabel95@gmail.com (B.L.); anacatarinasoaressousa@hotmail.com (A.C.S.); andrefmc17@gmail.com (A.C.); alexandra.rema@gmail.com (A.R.); mariamanuel.balca@gmail.com (M.B.); ataydelm@gmail.com (L.A.); cmmendonca@icbas.up.pt (C.M.); ruialvites@hotmail.com (R.A.); 2 Centro de Estudos de Ciência Animal (CECA), Instituto de Ciências, Tecnologias e Agroambiente da Universidade do Porto (ICETA), Rua D. Manuel II, Apartado 55142, 4051-401 Porto, Portugal; 3 Associate Laboratory for Animal and Veterinary Science (AL4AnimalS), 1300-477 Lisboa, Portugal; 4 Campus Agrário de Vairão, Centro Clínico de Equinos de Vairão (CCEV), Rua da Braziela n° 100, 4485-144 Vairão, Portugal; 5 3B’s Research Group, I3Bs—Research Institute on Biomaterials, Biodegradables and Biomimetics, University of Minho, Headquarters of the European Institute of Excellence on Tissue Engineering and Regenerative Medicine, AvePark, Parque de Ciência e Tecnologia, Zona Industrial da Gandra, Barco, 4805-017 Guimarães, Portugal; lucilia.silva@i3bs.uminho.pt (L.P.d.S.); cristiana.costa@i3bs.uminho.pt (C.C.); apmarques@i3bs.uminho.pt (A.P.M.); 6 ICVS/3B’s—PT Government Associated Laboratory, 4805-017 Guimarães, Portugal; 7 Departamento de Patologia e Imunologia Molecular, ICBAS—School of Medicine and Biomedical Sciences, University of Porto (UP), Rua de Jorge Viterbo Ferreira 228, 4050-313 Porto, Portugal; iamorim@ipatimup.pt; 8 Institute for Research and Innovation in Health (i3S), Universidade do Porto, Rua Alfredo Allen 208, 4200-135 Porto, Portugal; 9 Institute of Molecular Pathology and Immunology, University of Porto (IPATIMUP), Rua Júlio Amaral de Carvalho 45, 4200-135 Porto, Portugal; 10 Cooperativa de Ensino Superior Politécnico e Universitário (CESPU), Avenida Central de Gandra 1317, 4585-116 Gandra, Portugal; 11 Finao Biotech Lda, Campus Politécnico 10, BioBIP, 7300-555 Portalegre, Portugal; fbatista@finaobiotech.com (F.B.); fmata@finaobiotech.com (F.M.); jrtransmontano@aol.com (J.T.)

**Keywords:** biomaterials, gellan gum, honey, hyaluronic acid, regenerative medicine, skin regeneration, wound healing

## Abstract

**Background/Objectives**: Chronic wounds pose a significant health concern, with their prevalence increasing due to various etiologies. The global aging population further contributes to this rise, placing a substantial burden on healthcare systems in developed countries. This work aimed to develop new therapeutic options in the form of creams and dressings based on honey, gellan gum, and hyaluronic acid for preventing and treating chronic wounds across all stages. **Methods**: To address this, after the formulations were developed, in vitro cytocompatibility was determined. To confirm biocompatibility, an ovine wound model was used: full-thickness excisional wounds were treated with three formulations, namely gellan gum and honey sponges (GG-HNY), gellan gum, honey and hyaluronic acid sponges (GG-HA-HNY) and a honey-based cream (cream FB002). Daily assessments, including visual evaluation and wound scoring, were conducted for 30 days. Following the study period, tissues were collected for histological analyses. **Results**: The macroscopic examination revealed that all therapeutic groups facilitated lesion closure. Lesion size reduction, granulation tissue disappearance, and scar tissue development were consistent across all groups, with the group receiving cream demonstrating an advanced stage of healing. All groups achieved substantial wound closure by day 30, with no significant differences. Histopathological analysis following ISO standards revealed that GG-HA-HNY had the lowest ISO score, indicating minimal reactivity and inflammation, which corroborated the cytocompatibility. **Conclusions**: Combining these insights with previous findings enhances our understanding of wound regeneration dynamics and contributes to refining therapeutic strategies for chronic wounds. The formulations were designed to balance therapeutic efficacy with cost-effectiveness, leveraging low-cost raw materials and straightforward production methods.

## 1. Introduction

**Figure 1:**
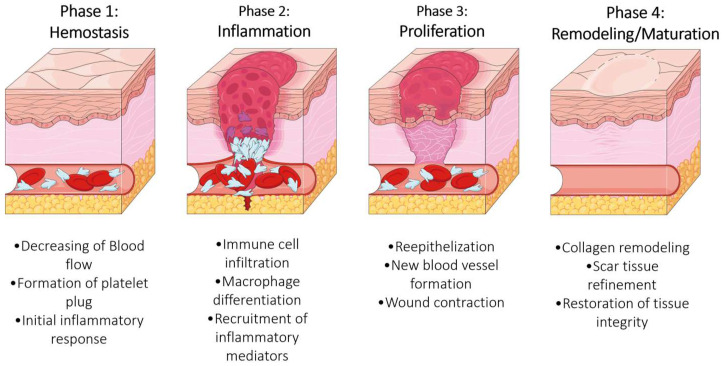
Schematic representation of the wound healing process. Based on [4] and generated and adapted for this paper using Servier Medical Art, provided by Servier, licensed under a Creative Commons Attribution 4.0 Unported License (Servier; https://smart.servier.com/, accessed on 7 February 2025).

The skin serves as the body’s primary defense against external aggressions, acting as a barrier against physical, chemical, and microbial threats. It plays a crucial role in temperature regulation, nervous sensitivity, and maintaining overall homeostasis. In normal conditions, the skin’s regenerative process is a highly coordinated and intricate series of events involving four key phases: hemostasis, inflammation, proliferation, and remodeling—Figure 1. During the hemostasis phase, the body immediately responds to injury by constricting blood vessels and forming a blood clot to prevent excessive bleeding. This is followed by the inflammation phase, where immune cells migrate to the wound site to combat potential infections and clear away debris. The proliferation phase is marked by the growth of new tissue and the formation of new blood vessels, while epithelial cells migrate across the wound bed to restore the skin barrier. Finally, the remodeling phase involves the maturation and reorganization of collagen fibers to strengthen the newly formed tissue and restore the skin’s integrity [1,2]. This intricate process is often associated with challenges such as scar tissue formation, infections, chronic wounds, and impaired tissue regeneration, including the development of keloids or hypertrophic scars [3].

Nevertheless, the regenerative process can be affected by several factors, leading to impaired tissue regeneration and prolonged wound healing. When the healing process is hindered, wounds can become chronic. Chronic wounds are defined by prolonged inflammation, lasting from 4 to 12 weeks, and are often complicated by infections, the formation of microbial biofilms, and insufficient responses from epithelial cells. These wounds are complex and multifactorial, frequently arising in individuals with underlying conditions, such as diabetes, infections, and arterial or venous insufficiency [5,6].

The prevalence of chronic non-healing wounds is on the rise, driven by factors such as population aging, age-associated diseases, concurrent health issues, tumors, and congenital defects. This trend negatively impacts the quality of life for millions globally, contributing to an escalating socioeconomic and healthcare burden. Current therapies, encompassing debridement, antibiotherapy, and dressings, fall short in terms of efficacy, making the exploration of novel treatments essential [4,5,6].

Hydrogels hold significant promise for enhancing wound healing due to their multifunctional properties. They offer biocompatibility, maintaining tissue homeostasis without damaging local tissue, and biodegradability, providing a temporary scaffold during the crucial phases of wound healing, such as fibroblast proliferation, re-epithelialization, and neovascularization. The bioadhesive nature of hydrogels ensures long-term stability around the wound, maintaining a moist environment and absorbing exudates, which is essential for effective healing. Furthermore, hydrogels can be engineered with antimicrobial properties to prevent infections, anti-inflammatory properties to expedite the transition from the inflammatory to the proliferation stage, and pro-angiogenic properties to promote angiogenesis, enhancing oxygen and nutrient delivery to the wound bed. The incorporation of drugs or therapeutic agents into hydrogels allows for controlled and sustained release, responding to environmental stimuli and further improving their therapeutic efficacy in chronic wound healing [7,8].

Gellan gum (GG)-based spongy-like hydrogels, resulting from GG-based hydrogels after a freeze-drying and re-hydration process, present biological, physico-chemical, and mechanical features that make them interesting to be used in cutaneous wound treatment. They present a wide microarchitecture that allows the absorption of high-water amounts, which is crucial for both absorbing the wound exudate and keeping the wound moist. Furthermore, spongy-like hydrogels hold enhanced resilience to deformation, allowing easy accommodation to the wound site and presenting off-the-shelf storage that facilitates their use under clinical practice. Hyaluronic acid (HA) integrated within the core GG-based spongy-like hydrogels provides additional biological activity owing to HA fragments resulting from hyaluronidases-mediated degradation. Importantly, HA concentration capable of improving angiogenesis was previously identified. Furthermore, both acellular and cellular GG-HA spongy-like hydrogels were shown to improve the wound healing process by accelerating wound closure, improving matrix remodeling, neovascularization, and neoinnervation [9,10].

The wound healing process can be significantly enhanced by natural products with medicinal properties [11]. Among these, honey (HNY) has gained attention due to its diverse bioactive components, including sugars, amino acids, enzymes, lipids, organic acids, carbohydrates, vitamins, flavonoids, phenolic acids, and minerals. These constituents contribute to honey’s well-documented antioxidant, anti-inflammatory, antibacterial, anti-nociceptive, and analgesic properties [12]. Scientific evidence from both preclinical and clinical studies has demonstrated that honey accelerates the healing of various wound types, including burns and surgical wounds [11]. Despite its numerous therapeutic benefits, it is essential to acknowledge that honey may trigger allergic reactions in certain individuals. These allergic responses are primarily linked to trace amounts of pollen, bee-derived proteins, or other natural contaminants. Symptoms can range from mild manifestations, such as itching, swelling, or hives, to severe cases of anaphylaxis. Individuals with pollen allergies, bee venom hypersensitivity, or a history of severe allergic reactions should exercise caution when consuming honey or applying it topically. As with any natural product, awareness of potential allergens is crucial to ensuring its safe and effective use [13].

To further explore the therapeutic potential of honey in wound healing, animal models serve as valuable tools for evaluating the efficacy and biocompatibility of new formulations. These models provide a controlled environment to simulate human wound healing processes, allowing researchers to investigate the interactions between the wound bed and applied therapies [14].

In this context, full-thickness wounds in a mice model treated with GG-HA-HNY spongy-like hydrogels exhibited improved epidermis thickness. Additionally, these wounds showed a tendency for higher accumulation of pro-inflammatory mediators (TNF-α and IL-6) and angiogenic factors (FGF-b and IGF-1) compared to the sham group. These findings suggest that the hydrogel formulation influences key wound healing mechanisms, including re-epithelialization, inflammation, and angiogenesis [15].

While rodent models provide valuable insights into wound healing mechanisms, their physiological differences from humans can limit the direct translation of findings to clinical applications. To bridge this gap, the ovine animal model has emerged as a reliable and suitable candidate for studying various disease models, including wound healing, due to its closer anatomical and physiological resemblance to human skin. Their cost-effectiveness, abundant availability, and minimal housing requirements make them highly practical choices for research. Additionally, sheep and goats are known for their docile temperament, ease of handling, and low maintenance costs, further supporting their suitability for experimental studies. Compared to other domestic species, such as dogs and non-human primates, they also receive greater ethical and social acceptance in research settings. Various research groups have successfully employed these animal models across multiple disciplines, demonstrating their relevance in biomedical studies. However, while these investigations provide essential insights, further extensive and targeted research is often required to facilitate seamless translation to human applications. Given their numerous advantages, small ruminants hold great potential as robust and translational animal models, particularly in regenerative medicine. In the context of wound healing, where effective treatments must promote rapid healing, alleviate pain, and restore normal tissue function, sheep and goats present promising prospects. Their large anatomical skin surface area and manageable disposition make them ideal candidates for studying novel wound healing therapies. Several research groups have already explored the regenerative potential of various treatments in these animals, employing interventions such as gels and Platelet-Rich Plasma. These studies highlight the potential of small ruminants as valuable models for advancing wound healing and regenerative medicine. By leveraging their unique advantages, future research using these models could contribute significantly to the development of innovative treatments with direct benefits for human healthcare [3,16,17,18].

This work aimed to develop and formulate new therapeutic options in the form of creams and dressings based on HNY, GG, and HA. While the individual components have been previously studied for wound healing, our research introduces an innovative approach by integrating them into a spongy-like hydrogel formulation designed to maximize their synergistic effects. The goal was to test the efficacy of these formulations in preventing and treating chronic wounds at all stages of the healing process. Building on the previous successful studies in rat models, this research expands its scope to sheep models to better replicate human skin physiology and enhance the clinical relevance of the findings. This was achieved through in vitro studies to characterize the formulations and assess their properties, followed by application in in vivo models of skin regeneration to evaluate their therapeutic potential. By combining the antimicrobial, anti-inflammatory, and pro-regenerative properties of HNY, GG, and HA into a single formulation, this study offers a novel therapeutic strategy aimed at improving wound healing outcomes and addressing the persistent challenges associated with chronic wounds.

## 2. Materials and Methods

### 2.1. Formulations

GG-HNY and GG-HA-HNY spongy-like hydrogels were prepared according to a methodology patented and published by the group [19] and optimized to include the honey. Gelzan (Sigma, Darmstadt, Germany) was dissolved in deionized H_2_O (0.25% (*w*/*v*)) under stirring at 90 °C for 30 min and then cooled to room temperature. HA (1.3-1-8 MDa, Lifecore, Chaska, MN, USA) was dissolved in deionized H_2_O (0.25% (*w*/*v*)) at room temperature under stirring for 3 h. Both solutions were filtered using a 0.22 µm filter, frozen at −80 °C, and freeze-dried (LyoAlfa 10/15, Telstar, Madrid, Spain) for 7 days to obtain sterile GG and HA. HNY (Monte Novo, Portalegre, Portugal) was sterilized by beta rays (accelerated electron beams). GG-HNY and GG-HA-HNY spongy-like hydrogels were prepared in sterile conditions under a flow chamber. Accordingly, gelzan was dissolved in sterile deionized H_2_O (1.25% (*w*/*v*)) under stirring at 90 °C for 30 min. For the preparation of hydrogels containing HA, HA (0.75% (*w*/*v*)) was dissolved in sterile deionized H_2_O at RT under stirring for 3 h prior to the addition of the GG. After dissolution, the temperature was reduced to 45 °C, and the HNY (30% (*v*/*v*)), pre-heated at the same temperature, was added under stirring. After homogenization, the solutions were poured into molds (3 × 2 × 3 cm, width × length × height). Hydrogels were progressively formed until the RT was reached. Spongy-like hydrogels were prepared from these hydrogels after osmotic stabilization in phosphate-buffered saline (PBS) solution (Sigma, Burbank, CA, USA) for 2 h, freezing at −80 °C and freeze-drying (LyoAlfa 10/15, Telstar, Spain) for 3 days. Cream FB002 was sterilized through a process involving pulsed electric fields and a predetermined sequence of ultrasound waves. Due to the proprietary nature of the patent, further details about the process cannot be disclosed.

### 2.2. In Vitro Assays

#### 2.2.1. Cell Culture

Mouse fibroblasts—L929 cell line (Merk^TM^, 85011425, Rahway, NJ, USA) were cultured in Dulbecco’s Modified Eagle Media—DMEM (Gibco^®^; 21885-025, Grand Island, NY, USA) and supplemented with 10% (*v*/*v*) fetal bovine serum—FBS (Gibco^®^; A31608-01), 1% (*v*/*v*) Penicillin–Streptomycin (Gibco^®^; 15140122), 1% (*v*/*v*) Amphotericin B (Gibco^®^, 15290026), and 1% (*v*/*v*) HEPES buffer solution (Gibco^®^, 15630122). L929 cells were maintained at 37 °C in a humidified atmosphere with 95% atmospheric air and 5% CO₂.

#### 2.2.2. Cytocompatibility

For the cell viability assay regarding the interaction between L929 cells and the cream and GG-HNY and GG-HA-HNY dressings, the PrestoBlue^TM^ reagent was used. This reagent is a water-soluble solution based on resazurin (7-hydroxy-3H-phenoxazine-3-one-10-oxide) that is commercially available and ready to use [20]. Living cells reduce this compound to resorufin at the mitochondrial level, a process accompanied by a change in the color and fluorescence of the solution. Thus, the absorbance values of the solution serve as an indicator of cell viability, allowing the quantitative measurement of cell proliferation.

Both GG-HNY and GG-HA-HNY sponges were evaluated in a direct cytotoxicity assay. L929 cells were seeded at a density of 8000 cells/cm^2^ in wells of a 24-well plate where representative fragments of sponges had previously been placed. The set of L929 cells and sponge fragments were then covered with the culture medium described above. Four groups were considered in this assay: GG-HNY, GG-HA-HNY, DMEM 10% (consisting of L929 cells seeded in the wells of the 24-well plate in standard culture medium and with no contact with any biomaterial, serving as a negative control), and DMSO 10% (in which FBS supplementation in the culture medium was replaced with a proven toxic agent, Dimethyl sulfoxide—DMSO, serving as a positive control). For each group, quadruplicates for each test group with cells and 2 wells without cells were considered blanks. The plates were maintained under standard culture conditions throughout the assay (37 °C, 5% CO_2_). At each evaluation timepoint (24 h (1 day), 72 h (3 days), 120 h (5 days), and 168 h (7 days)), the culture medium was removed from each well, and fresh complete medium was added to each one, with 10% of the cell viability reagent PrestoBlue^TM^ (Invitrogen^®^, A13262, Waltham, MA, USA). The cells were then incubated for 1 h at 37 °C in 5% CO_2_.

For the FB002 cream, due to the viscous nature of the cream under evaluation, which prevented the direct cytotoxicity assay, an indirect contact assay was chosen. In this assay, the tested cream was placed inside an insert (approximately 1 mL of cream in each insert), while L929 cells were seeded at a density of 8000 cells/cm^2^ in wells of a 24-well plate covered with the respective culture medium. The inserts were then placed inside each well in such a way that the cream came into contact with the standard culture medium through the porous surface of the insert, allowing the diffusion of compounds of the cream into the medium and their indirect contact with the cultured cells. Three groups were considered in this assay: cream FB002, with the characteristics described above; DMEM 10% (consisting of L929 cells seeded in the wells of the 24-well plate in standard culture medium, without contact with cream, serving as a negative control) and DMSO 10% (in which FBS supplementation in the culture medium was replaced with a proven toxic agent, DMSO, serving as a positive control). For each group, quadruplicates for each test group with cells and 2 wells without cells were considered blanks. The plates were maintained under standard culture conditions throughout the assay (37 °C, 5% CO_2_). At each evaluation timepoint (24 h (1 day), 72 h (3 days), 120 h (5 days), and 168 h (7 days)), the culture medium was removed from each well, and fresh complete medium was added to each one, with 10% of the cell viability reagent PrestoBlue^TM^ (Invitrogen^®^, A13262). The cells were then incubated for 1 h at 37 °C in 5% CO_2_.

Changes in cell viability were detected by absorbance spectroscopy using the Thermo Scientific Multiskan FC spectrophotometer. The supernatant was collected and transferred to a 96-well plate, and absorbance was read at 570 nm and 595 nm. For each well, the absorbance at 595 nm (normalization wavelength) was subtracted from the absorbance at 570 nm (experimental result). Corrected absorbance was obtained by subtracting the Mean of control wells for each experimental well.

Subsequently, the seeded wells were washed with phosphate buffer solution, DPBS (Gibco^®^, 14190-144), to remove any residues of PrestoBlue^TM^, and then fresh culture medium was replenished in each well.

### 2.3. Biocompatibility and Preclinical Trials

#### 2.3.1. Animals and Ethical Issues

The detailed description of all activities to be carried out in the ovine model was submitted to the Animal Welfare Body (ORBEA) Instituto de Ciências Biomédicas Abel Salazar of Porto University (ICBAS-UP) through a dossier containing all the procedures to be carried out on the animals, justification of their need, measures taken to minimize the number of animals used and to avoid and to reduce discomfort and suffering. Project approval (project no. 407/2021/ORBEA) allowed the implementation of planned in vivo activities for the ovine model.

All activities were conducted in accordance with EU Directive 2010/63/EU and its transposition into Portuguese law, following the principles outlined in the OECD document “Guidance Document on the Recognition, Assessment, and Use of Clinical Signs as Humane Endpoints for Experimental Animals Used in Safety Evaluation (2000)”. Additionally, all measures were taken to avoid or reduce any pain or discomfort in the animals, considering human indicators for identifying stress and animal suffering.

#### 2.3.2. Testing Formulations in a Critical Skin Lesion Model in Sheep

Four female Merino sheep (Ovis Aries), weighing between 30 and 40 kg, were acquired from a certified breeder, previously approved by the hosting institution, and with a disease-free status (B3) or officially free (B4) from brucellosis. The sample size was determined by practical and ethical considerations, such as minimizing animal use while still achieving biologically relevant outcomes. No animals, experimental units, or data points were excluded from the analysis. The animals underwent pre-movement tests for the detection of infectious diseases. Additionally, all animals were tested and vaccinated for bluetongue. Upon arrival, all animals underwent a quarantine and acclimatization period of fifteen days and were subjected to a prophylactic protocol, including corrective hoof trimming, shear, internal deworming, and vaccination against enterotoxemia. Before being surgically intervened, and regularly throughout the study, the animals underwent a general physical examination. The animals were maintained in communal groups to ensure the expression of their gregarious behavior, were fed with hay and concentrate according to their nutritional needs, and had continuous access to fresh water. Lesions were randomly assigned to treatment groups by random draw. Blinding was implemented during treatment application, outcome assessment, and data analysis to minimize bias.

For the surgical procedure, the animals fasted for 12 h and were then sedated with 0.2 mg/kg of Butorphanol (Alvegesic^®^, InoVet, Arques, France) and 0.2–0.3 mg/kg of Xylazine (Rompun^®^, Bayer, Leverkusen, Germany) intramuscularly. The dorsum was properly shaved and cleaned to guarantee the aseptic conditions of the procedures. Subsequently, anesthesia induction was performed using tiletamine–zolazepam (Zoletil^®^, Virbac, Carros, France), 6.6 mg/kg, intravenously.

**Figure 2:**
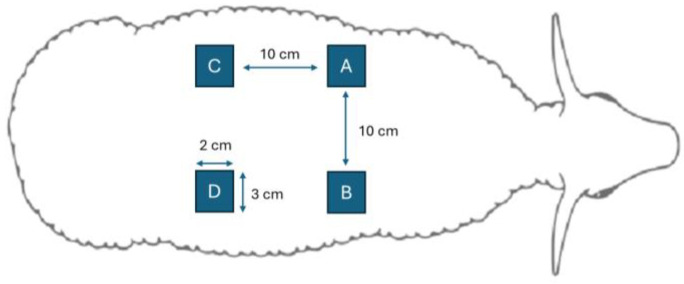
Schematic representation of the lesions created on the back of each animal and the respective applied treatments. A—Sham: control; B—GG-HNY: gellan gum and honey; C—GG-HA-HNY: gellan gum, honey, and hyaluronic acid; D—cream: cream FB002.

Four full-thickness skin lesions (critical lesions) with dimensions of 3 cm × 2 cm (6 cm^2^) were created on the back of each animal (Figure 2), separated by 10 cm. No treatment was applied to one of the lesions—the control lesion (sham). HNY-based spongy formulations were applied to two lesions—one consisting of GG and HNY (GG-HNY) and the other consisting of GG, HA, and HNY (GG-HA-HNY). In the fourth and final lesion, the cream FB002 was applied (cream). After inducing the lesions and applying the respective therapeutic approaches, the animals’ backs were covered with a dressing adapted for protection against environmental contamination, consisting of applying gauze compresses with petroleum jelly. Finally, anti-inflammatory (Meloxicam—Meloxidyl^®^, Ceva, Marseille, France, 0.5 mg/kg IM) and prophylactic antibiotherapy (Penicillin–Streptomycin—Lilimicina^®^, Ceva, 24,000–66,000 IU/kg) were applied every 72 h for a total of 3 administrations. In the first week after surgery, to reduce the risk of wound contamination, the animals were kept in individual metabolic cages and then returned to their communal group. Their dressings were changed every other day.

##### Evaluation of Cutaneous Regeneration

After the surgical intervention and respective therapeutic approaches, all animals and their lesions were examined and photographed every 4 days for a month to monitor the scarring and regenerative profile of the lesions. To avoid bias in the macroscopic analysis, the same examiners were used throughout the study. These records were subsequently used for macroscopic analysis, particularly in determining the percentage of closure, lesion area, and wound scoring quantification.

##### Macroscopic Description of Skin Lesions

Skin lesions were macroscopically evaluated over a period of 4 weeks, concurrently with photographic documentation. To properly assess the evolution of each lesion based on inflammatory parameters and dimensional characteristics of the wound, the parameters indicated in Table 1, adapted from Holzer-Geissler et al. 2022 [21], were used.

The final score for each lesion was calculated based on the inflammatory and lesion area scores. Inflammatory parameters were based on the presence of purulent secretions and swelling, erythema, and its respective extension. For lesion area assessment, the following parameters were included: lesion dimensions, moisture in the lesion, presence of granulation tissue, scab formation, and necrosis. A score was assigned to each parameter based on the lesion phenotype, as described in Table 1. Inflammatory parameters were assigned a maximum score of 8, and lesion area evaluation parameters a maximum of 13. Each wound could thus reach a maximum score of 21 at each assessment time.

On the first day of the study, each lesion was classified with a score of 7, as the surgery required full-thickness excision of the skin.

##### Determination of Closure Percentage and Lesion Area

After photographic records at each timepoint, lesion photos underwent a digital assessment to determine the parameters “closure percentage” and “lesion area” of complementary significance. The evaluation was performed using ImageJ^®^ software (version: 2.3.0/1.53q).

For each lesion, length and width were measured, and the lesion area was obtained using the following formula (Equation (1)):

(1) $$Lesion\;Area\;\left({\mathrm{cm}}^{2}\right)=width\times lenght\;$$

At each timepoint, the lesion closure percentage was calculated using the following formula (Equation (2)):

(2) $$Closure\;Percentage\;\left(\%\right)=\frac{Initial\;Area-Final\;Area}{Inicial\;Area}\times 100.\;$$

where the Initial Area is the area calculated immediately after the surgical intervention (closure percentage of 0%), and the Final Area is the area calculated at each timepoint.

##### Histopathological Evaluation

After the 30-day study period, skin biopsies with a diameter of 10 mm, full thickness, including part of the healed lesions in each therapeutic group and adjacent healthy skin, were collected. The biopsy was preceded by sedation with Butorphanol (Alvegesic^®^, VMD nvInoVet) and Xylazine (Rompun^®^, Bayer) via intramuscular injection. The region around the lesions was trichotomized and subjected to rigorous cleaning and asepsis, and biopsies were collected using a 10 mm diameter biopsy punch. Tissue samples were fixed with 4% formaldehyde (Merck^TM^; 100496), and an assessment of local reactivity phenomena was also performed during collection. After the biopsy, new lesions were sutured with absorbable suture thread, and the animals returned to their usual communal housing.

The collected samples were processed with Hematoxylin–Eosin (H&E) staining in 3 mm thick sections for subsequent histopathological analysis. The evaluation of sections was performed using a Nikon Microscope (Nikon, Amstelveen, The Netherlands, Eclipse E600) connected to a camera (Nikon, Amstelveen, The Netherlands, Digital Sight DS-5M) for image recording. The samples were assessed by an experienced Veterinary Pathologist, and the histological organization of the skin achieved after the regenerative process, levels of fibrous tissue infiltrated in scars, and the presence of phenomena such as neovascularization, fibrosis, infiltration of inflammatory cells, necrosis, fat infiltration, calcification, foreign body reactions, presence of granulomas, among others, around the lesion site were determined. This evaluation was performed following the assumptions of ISO-10993-6:2016 [22], Annex E. Each sample was assessed and classified according to individual parameters and using a semi-quantitative classification. The samples were classified as showing “absence of reaction or minimal reaction” (classification from 0 to 2.9), “mild reaction” (classification from 3 to 8.9), “moderate reaction” (classification from 9 to 15), or “severe reaction” (classification > 15).

##### Statistical Analysis

Statistical analysis was performed using the GraphPad Prism software version 9.00 for Windows (GraphPad Software, La Jolla, CA, USA). Whenever appropriate, data and results were expressed as Mean ± SEM. Comparisons between groups in the results of different tests are based on the application of a parametric test. A two-way ANOVA was used to analyze the data, allowing for the evaluation of the interaction between independent variables. A value of *p* < 0.05 was considered statistically significant. The significance of the results is shown according to *p*-values by the symbol *: * corresponds to 0.01 ≤ *p* < 0.05, ** to 0.001 ≤ *p* < 0.01, *** to 0.0001 ≤ *p* < 0.001, and **** to *p* < 0.0001.

## 3. Results and Discussion

### 3.1. Cell Viability Assay

**Figure 3:**
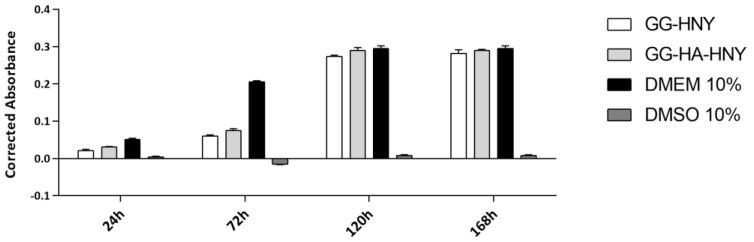
Corrected absorbance assessed by the PrestoBlue^TM^ viability test for direct contact between L929 cells, GG-HNY, and GG-HA-HNY, up to 168 h. Results presented as Mean ± SEM.

**Figure 4:**
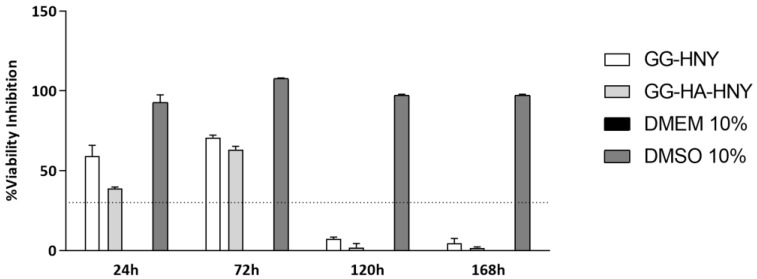
Percentage of cell viability inhibition after the direct contact of L929 cells with GG-HNY and GG-HA-HNY up to 168 h. Results presented as Mean ± SEM. The dashed line represents the percentage of cell viability inhibition above which cytotoxicity is considered, according to ISO 10993-5:2009 [23], (30%).

The results of the cytocompatibility between L929 in the GG-HNY and GG-HA-HNY samples can be found in Figure 3 and Table S1 and Table 2. Additionally, the percentage of inhibition of cell viability, normalized to the control group, was also determined, as shown in Figure 4 and Table S2.

The results show that, similarly to what was observed in the negative control, over time, there is an increase in cell viability for both materials to be evaluated, GG-HNY and GG-HA-HNY. At 24 h, marked statistical differences are recorded between these two groups and the negative control DMEM 10%, differences that disappear in the last timepoints, and at 168 h, the viability values observed in the three groups are similar, without statistical differences. The viability values associated with the positive control, DMSO 10%, are, as expected, low due to the known cytotoxic effect of this compound.

When evaluating the % cell viability inhibition, using the negative control group as a standard, it is worth noting that in the first two timepoints evaluated, 24 and 72 h, both groups, GG-HNY and GG-HA-HNY, induce a viability inhibition greater than 30%, an effect that decreases greatly at the final timepoints, 120 h and 168 h, in which the toxic effect is below the threshold considered for the establishment of cytotoxicity according to ISO 10993-5:2009 (30%). These results allow us to establish that, under the presented test conditions, the compounds under study are cytotoxic in the acute phase, after initial contact with cells, probably inducing initial cell adhesion cell proliferation restrictions. This negative effect seems to disappear over time due to cellular adaptation mechanisms, making them suitable for application in long studies and in complex and time-consuming regeneration processes such as those of the skin. DMEM 10% consistently demonstrated a % of cellular inhibition greater than 30%, as expected.

**Figure 5:**
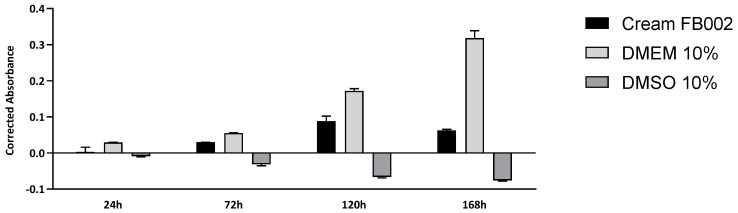
Corrected absorbance assessed by the PrestoBlue^TM^ viability test for indirect contact between L929 cells cream FB002, up to 168 h. Results presented as Mean ± SEM.

**Figure 6:**
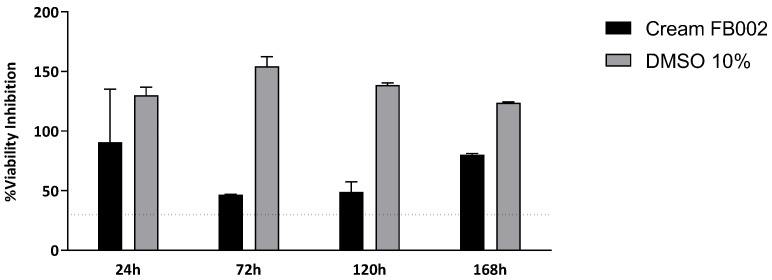
Percentage of cell viability inhibition after the indirect contact of L929 cells with the cream up to 168 h. Results presented as Mean ± SEM. The dashed line represents the percentage of cell viability inhibition above which cytotoxicity is considered, according to ISO 10993-5:2009 (30%).

The values for cytocompatibility between L929 cells and cream FB002 can be found in Figure 5 and Table S3 and Table 3. Additionally, the percentage of inhibition of cell viability, normalized to the control group, was also determined, as shown in Figure 6 and Table S4.

The results demonstrate that as early as 24 h, there is an inhibition of L929 cell adhesion in the cream and DMSO groups, with early cytotoxicity expected in the latter. At this early timepoint, statistical differences are observed only between the DMEM 10% and the DMSO 10% groups. The cell viability of the negative control group followed an expected progression, with a continuous increase over time. In the cream FB002 group, an increase in cell viability was also observed at 24 h and 120 h, but it was lower than in the control group, with statistical differences observed between the two groups in both timepoints. At 168 h, there was even a decrease in cell viability in cells subjected to contact with the cream, revealing that long-term contact increases cytotoxicity at later timepoints. In the DMSO 10% group, there was a progressive decrease in cell viability over the evaluation times, as expected.

The study of the percentage of cell inhibition indicates results of similar significance. At all timepoints of the cell viability study, using the control group as a standard, a percentage of inhibition higher than 30% was observed for both the cream FB002 group and the DMSO 10% group, at all timepoints. Considering that 30% inhibition is considered the cytotoxicity threshold, all compounds that exhibit a percentage of inhibition above this value at a specific timepoint are considered cytotoxic. These results allow us to conclude that under the presented test conditions, the cream FB002 is cytotoxic to L929 cells.

These assays were conducted to test cytocompatibility with L929 cells, murine fibroblasts that, in this case, mimic skin cells, a justifiable choice since the skin is the organ where this formulation is intended to be applied in vivo. In the case of sponges, it was possible to perform the cytocompatibility test by direct contact. Since the cream FB002 has a viscous consistency, tending toward liquid, it was impossible to perform a cytocompatibility assay by direct contact. Therefore, an adapted assay was chosen by indirect contact, in which cells were seeded at the bottom of the wells of a 24-well plate while the cream was placed inside a porous insert, allowing the dissolution of cream compounds in the culture medium and their contact with cells without the physical inhibition caused by the cream per se.

According to the assumptions followed in this assay and based on the principles advocated in ISO 10993-5:2009, the cream FB002 is considered cytotoxic, and the GG-HNY and GG-HA-HNY sponges are cytotoxic in the acute phase and cytocompatible in the chronic contact phase. It is important to note that a cytotoxicity assay assesses the influence of a compound on a specific cell line when cultured under specific conditions that can themselves influence the compound–cell interaction, and the identification of cytotoxicity in this assay should not be considered definitive and indicative of the absence of efficacy once the compound is applied in vivo. Specifically in the case of the FB002 cream, the observed viscous, tending toward liquid state in the cream may facilitate excessive dilution of its constituent compounds in the culture medium, saturating the medium and negatively affecting the cells. Modifying the physical characteristics of the cream may attenuate this effect and reduce the cytotoxic effect of the cream. This effect will not be felt as such in an in vivo application when the cream is directly applied to a wound.

### 3.2. Preclinical Trials in Ovine Model

#### 3.2.1. Macroscopic Description of Skin Lesions

**Figure 7:**
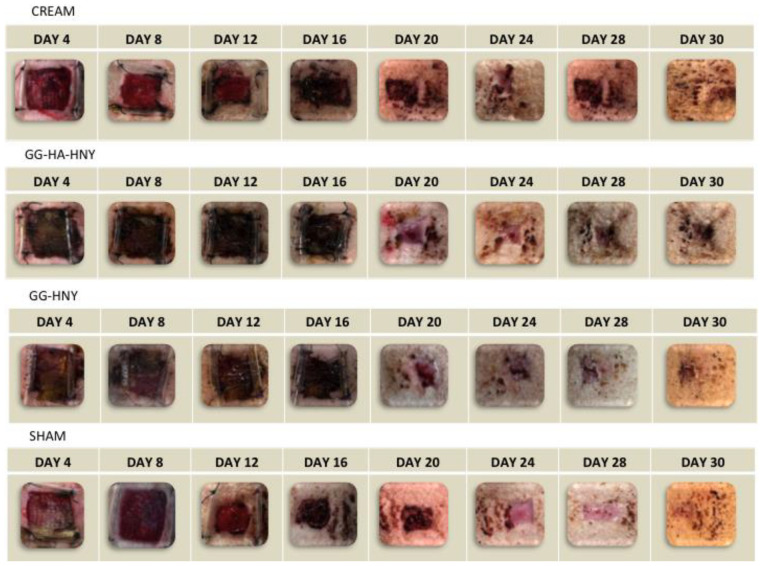
Visual representation of the macroscopic evolution of lesions subjected to different treatments over the 4 weeks of study.

The macroscopic evolution of the appearance of lesions with different treatments can be observed in Figure 7.

In the sham, GG-HNY, and GG-HA-HNY groups, no significant differences in size are observed until day 8. In the cream group, a reduction in lesion dimensions seems to be identified by day 8. From day 12 onward, a progressive reduction in lesion dimensions is observed in all groups, with nearly complete closure by day 28 and total closure by day 30. Until day 16, the groups receiving spongy formulations exhibited a darkened coloration unrelated to the lesion itself but associated with the dehydrated sponge, which presented the observed appearance until removed from the lesion site while the regenerative process occurred in depth. Both the sham and cream groups showed a healthy hemorrhagic bed as early as day 4, indicative of a correct healing process, resulting in a faster reduction in lesion dimensions for these groups. The macroscopic appearance was similar for all therapeutic groups by day 30.

**Figure 8:**
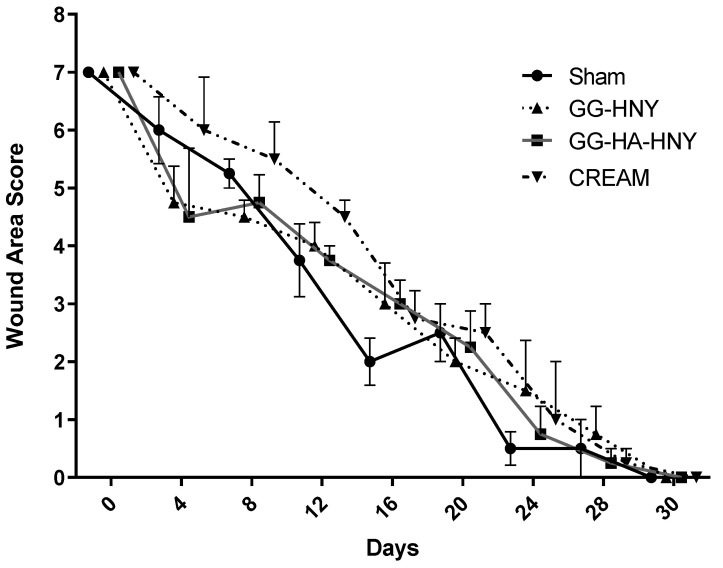
Wound Area Score over the study period. Results presented as Mean ± SEM.

The results of the lesion area scores can be observed in Figure 8 and Table S5.

The Wound Area Score is based on the assessment of parameters such as moisture in the lesion, lesion dimensions, presence of granulation tissue, scar formation, and presence of granulation tissue. After assigning the maximum value of 7 for this score immediately after the lesion, a continuous decrease in values was observed over time, with the progressive reduction in lesion dimensions during closure, disappearance of granulation tissue, and exuberant scar tissue observed in the early timepoints, and disappearance of evident moisture observed in the early days. No therapeutic group showed the presence of necrotic tissue over the study period. There were no statistically significant differences observed between any of the therapeutic groups at any of the considered timepoints.

**Figure 9:**
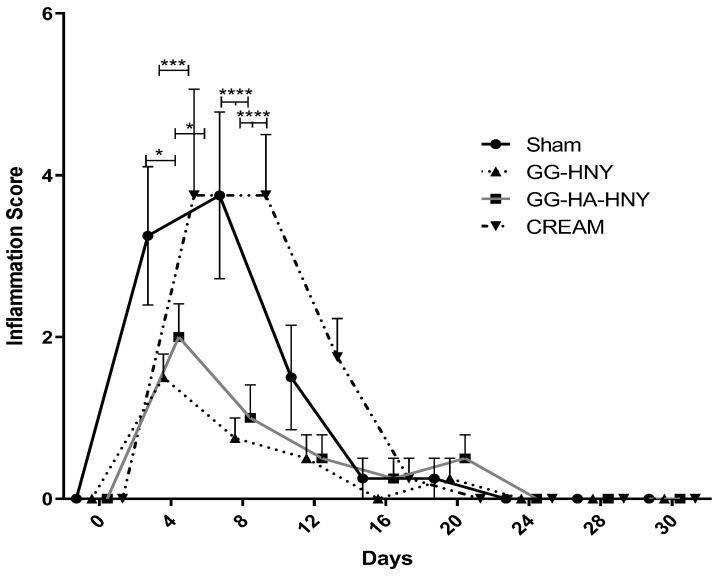
Inflammation score over the study period. Results presented as Mean ± SEM. Significance compared to the control is indicated according to the *p*-values with one, three, or four (*) symbols corresponding to 0.01 ≤ *p* < 0.05, 0.0001 ≤ *p* < 0.001, and *p* < 0.0001, respectively.

The results of the inflammation scores for the lesions can be found in Figure 9 and Table S6.

The inflammation score is based on the assessment of parameters such as purulent secretion, presence of erythema, extent of the region with erythema, and presence of edema and swelling—clinical indicators of an inflammatory process. After assigning a score of 0 immediately after the surgical intervention, it is possible to observe a peak of the inflammatory process occurring between days 4 and 8 after lesion induction in all therapeutic groups. This is followed by a sharp decrease in the following days, consistently showing an absence of inflammation from day 20 onward. As inflammation is the first of the three necessary phases for cutaneous healing, its occurrence immediately after lesion induction is expected. The established inflammation in the sham and cream groups is visibly more intense than in the groups that received the honey-based spongy formulation. Significant statistical differences are observed on day 4 between the sham group and the GG-HNY group (*p* = 0.0140) and between the cream group and the GG-HNY (*p* = 0.0008) and GG-HA-HNY (*p* = 0.0140) groups. Similarly, on day 8, significant differences are observed between the sham group and the GG-HNY and GG-HA-HNY groups (*p* < 0.0001) and between the cream group and the GG-HNY and GG-HA-HNY groups (*p* < 0.0001). These results support the macroscopic observation in which the sham and cream groups appear to be in a more advanced stage of healing on these days compared to the remaining groups.

#### 3.2.2. Determination of Wound Closure Rate

**Figure 10:**
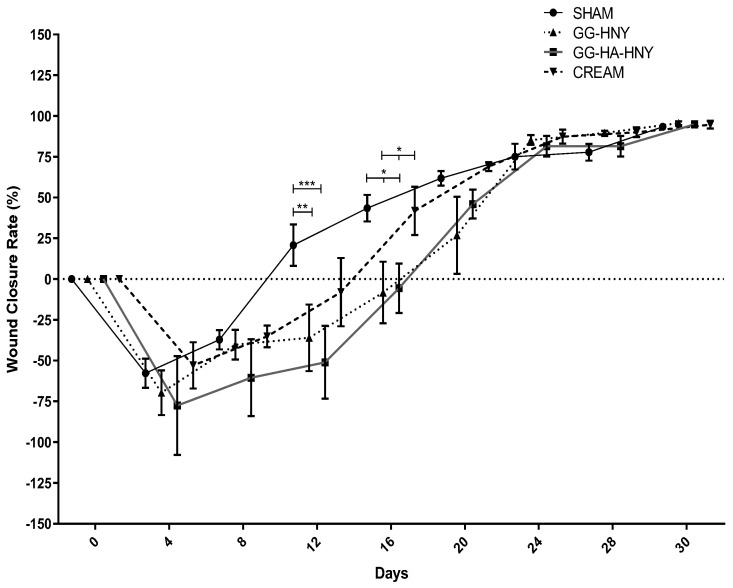
Wound Closure Rate (%) over the study period. Results presented as Mean ± SEM. Significance in comparison to the control is indicated according to the *p*-values with one, two or three (*) symbols corresponding to 0.01 ≤ *p* < 0.05, 0.001 ≤ *p* < 0.01, and 0.0001 ≤ *p* < 0.001, respectively.

The evolution of the Wound Closure Rate over the study period can be found in Figure 10 and Table S7.

In all therapeutic groups, a decrease in the closure rate was observed, graphically appearing as a negative trend. This phenomenon is related to a slight separation of the wound edges in the first days after induction, secondary to marked scar retraction typical in sheep. The group in which more pronounced scar retraction was observed is GG-HA-HNY, where the closure rate values were lower on day 4 after the lesion. From day 4 onward, a progressive increase in the closure rate of the lesions was observed in all groups, corresponding to the closure of the wounds. In all groups, a positive closure rate was observed, meaning a lesion of smaller dimensions than those induced surgically between day 16 and day 20. At the end of the study period, all therapeutic groups presented closure percentages of lesions between 93% and 95%. Statistically significant differences were observed between groups on day 12 and day 16, in the former between the sham group and the GG-HNY (*p* = 0.0073) and GG-HA-HNY (*p* = 0.0004) groups, and in the latter between the sham group and GG-HNY (*p* = 0.0177) and GG-HA-HNY (*p* = 0.0004), and between the cream group and GG-HNY (*p* = 0.0232) and GG-HA-HNY (*p* = 0.0349).

#### 3.2.3. Determination of Wound Area

**Figure 11:**
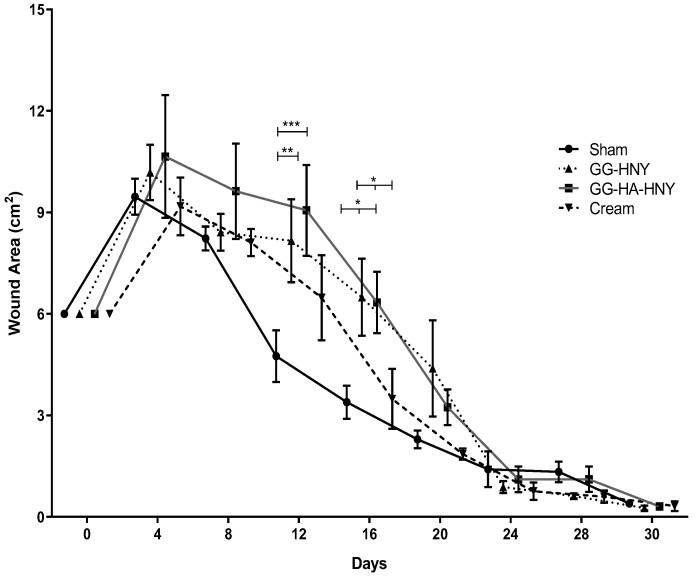
Evolution of the wound area over the study period. Results presented as Mean ± SEM. Significance in comparison to the control is indicated according to the *p*-values with one, two or three (*) symbols corresponding to 0.01 ≤ *p* < 0.05, 0.001 ≤ *p* < 0.01, and 0.0001 ≤ *p* < 0.001, respectively.

The values of the wound area over time can be observed in Figure 11 and Table S8 and are logically complementary to the Wound Closure Rate values. Due to the previously described scar retraction, in the early timepoints after inducing the lesions, area values higher than 6 cm^2^ of the original lesion were observed, with the highest area values reached by the GG-HA-HNY group. After day 4, there is a continuous decrease in the wound area for all therapeutic groups, reaching a nearly total closure by day 30, with an area very close to 0 cm for all groups. No statistically significant differences were observed between the groups after the 30-day follow-up, although differences were observed on days 12 and 16, equivalent to those seen in the previous graph.

In general, in vivo assays demonstrate that all treatments allow for cutaneous regeneration after inducing critical lesions, with a profile identical to that observed in the sham group. There is, therefore, no inhibition of normal cutaneous regeneration, although there is also no promotion of macroscopic regeneration, neither faster nor more effective than what occurs spontaneously.

In the macroscopic characterization of wounds, the cream group seems to perform better, with the lesion exhibiting a healthier appearance and characteristics like the control group. The appearance of groups receiving honey-based sponge formulations presents a physical limitation associated with the sponge itself, which, when dehydrated, appeared as a dried-out body over the lesions, hindering the observation of the regenerative process occurring beneath it. The lesion assessment scores, particularly the inflammation score, corroborate the macroscopic findings, with the cream group showing signs of a more intense post-surgical inflammation essential for the normal occurrence of the scarring process. The study of Wound Closure Rate and wound area demonstrated that all groups experienced an intense scar retraction phenomenon, delaying the closure process for a few days. However, this did not prevent the closure of almost all lesions 30 days after surgery.

Based on the data obtained, there were no observable or measurable macroscopic differences between the therapeutic groups that would establish any of them as more effective in promoting cutaneous regeneration after injury. Conversely, none of the therapeutic groups hindered the normal progression of the regenerative process. The groups with honey-based sponge formulations seemed to have experienced a slight delay in the initiation of the scarring process due to the physical presence of the dehydrated sponge and a less evident inflammatory process. However, after the 30-day study period, the results are similar for all groups.

#### 3.2.4. Histopathological Evaluation of the Cutaneous Regenerative Process

**Figure 12:**
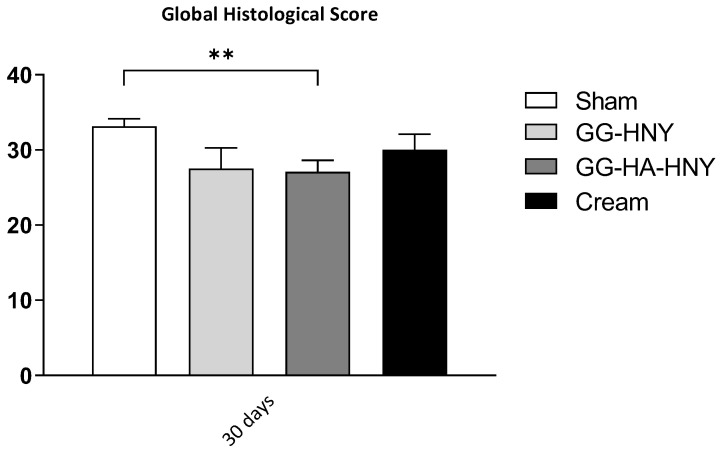
Histological Score Determination following ISO 10993-6:2016. The histological classification is presented as Mean ± SEM. Significance compared to the control is indicated according to the *p*-values with two (*) symbols corresponding to 0.001 ≤ *p* < 0.01.

**Figure 13:**
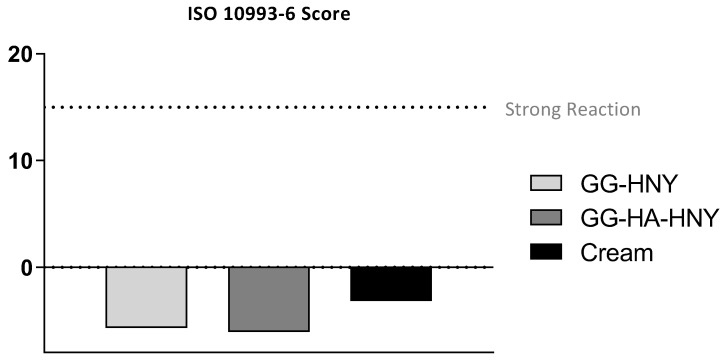
Classification of Lesion Sites following ISO 10993-6:2016. The dashed line indicates the classification above which the implant is considered to induce a severe reaction (ISO score > 15).

The results of the histopathological evaluation, following the assumptions of ISO ISO-10993-6:2016, Annex E, can be found in Figure 12 and Figure 13 and in Table S9.

**Figure 14:**
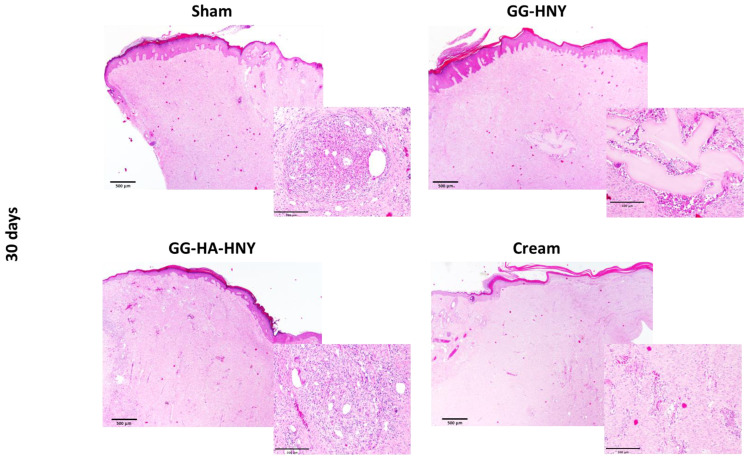
Histological evaluation of lesion sites after H&E staining. For each panel, the left image represents a 20× magnification (scale bar—500 µm), and the right image represents a 100× magnification (scale bar—100 µm).

At 30 days after lesion induction, the worst histopathological score was identified in the sham group, with better results observed in the therapeutic groups under study. The best result was obtained in the GG-HA-HNY group, where there were even statistically significant differences compared to the sham group (*p* = 0.0091). For the calculation of the final score, the results of the sham group were used as a control, and the overall histological score of this group was subtracted from the corresponding value of each therapeutic group. Thus, the ISO score of the sham group is indicated as zero, and the better performance of the remaining therapeutic groups results in negative ISO scores. Since the ISO 10993-6:2016 classification scale attributes an absence of reaction for scores up to 2.9, all established options can be classified as non-reactive. Representative images of the histological evaluation can be seen in Figure 14. In all groups, a variable inflammatory infiltrate is observed, particularly in the sham group. This infiltration is lower in the GG-HA-HNY group, where the therapeutic sponge seems to have inhibited the development of a more exuberant inflammatory reaction. The phenomena of neovascularization and fibrosis are transversal and comparable among the four considered groups. No lipid infiltration was observed in any therapeutic group. Despite the good histological results, the GG-HA-HNY group showed the presence of moderate-to-severe hemorrhagic phenomena. The observed hemorrhages may be secondary to a reaction to the structural constitution of the applied sponge.

Histopathological results do not fully corroborate the findings observed in the macroscopic evaluation. Histologically, although there was an apparent normal progression of the regenerative process in all groups, all therapeutic approaches promoted lower local reactivity compared to the sham group. Among the therapeutic options, the GG-HA-HNY group had the lowest ISO score, and therefore, it was the group with the least reactivity to the instituted treatment. It is also the group where, at 30 days, there is less infiltration of inflammatory cells, which may be a consequence of the lower inflammation score observed in this group at the initial timepoints during macroscopic evaluation. The apparent limitation due to the presence of the dehydrated sponge on the lesion site does not translate into regenerative limitations at the histological level. Despite the good performance of this group, including statistical differences compared to the sham group, the observed hemorrhagic phenomena and probable vasculitis in different animals should be considered. Once again, the cream group shows positive therapeutic performance, with no statistical differences compared to the sham group, reinforcing the need to modify the physical characteristics of the formulation for in vitro evaluation.

The results of histopathological evaluation confirm that no therapeutic options prevent the normal sequence of cutaneous regeneration, establishing the GG-HA-HNY treatment as the least reactive after application to the lesion site. The not entirely consistent results between macroscopic and histological evaluations reinforce the need for cutaneous regeneration assessment to be conducted on both scales, macro and microscopic, to ensure a return to normal tissue organization and function.

## 4. Conclusions and Further Directions

The comprehensive evaluation of different therapeutic approaches for cutaneous regeneration following critical lesions provides valuable insights into the potential applications and limitations of each treatment modality. The macroscopic assessment of lesion evolution, coupled with in-depth histopathological analysis, offers a nuanced understanding of the regenerative process.

The macroscopic examination revealed that all therapeutic groups facilitated the closure of induced lesions, with the cream group exhibiting a particularly healthy appearance similar to the sham group. Interestingly, groups receiving honey-based sponge formulations exhibited a temporary darkening, attributed to the presence of the dehydrated sponges, indicating a potential limitation in visualizing the regenerative process.

In terms of lesion scores, the reduction in size, granulation tissue disappearance, and scar tissue development were consistent across all therapeutic groups, indicating the efficacy of each approach. Notably, the cream group demonstrated a seemingly advanced stage of healing, supported by the rapid reduction in lesion dimensions and the presence of a healthy hemorrhagic bed.

Further examination using scores for inflammation highlighted differences among groups, with the cream and sham groups displaying more intense early inflammation. However, statistical significance in inflammation scores suggests that the honey-based sponge formulations may contribute to a milder inflammatory response, potentially facilitating a smoother healing process.

The evaluation of closure percentage and lesion area demonstrated a common initial setback due to scar contraction, impacting closure rates. Despite this, all groups achieved substantial closure by day 30, with no statistically significant differences among them. The cream group exhibited positive trends, aligning with the macroscopic observations.

Histopathological analysis, following ISO standards, provided a deeper understanding of tissue reactions to the treatments. The GG-HA-HNY group stood out with the lowest ISO score, indicating minimal reactivity and inflammation, results corroborated by the in vitro cytocompatibility assay performed. These results were statistically relevant when compared to the sham group. The cream group, while not statistically different from the sham group, showed positive therapeutic effects histologically, emphasizing the need for a nuanced interpretation of results across macroscopic and histological scales.

Despite overall positive outcomes, the HNY-based sponge formulations, especially GG-HA-HNY, raised concerns due to observed hemorrhagic phenomena and vasculitis in some cases. This underscores the importance of balancing therapeutic efficacy with potential adverse effects.

In conclusion, this study contributes valuable data on the macroscopic and histological aspects of cutaneous regeneration after different therapeutic interventions. The cream formulation demonstrated promising results, warranting further exploration and potential formulation refinements. The HNY-based sponge formulations, while effective, necessitate careful consideration of observed adverse effects. This comprehensive analysis sets the stage for refining and optimizing therapeutic strategies to enhance cutaneous regeneration outcomes in a one health approach. Future studies may explore direct comparisons with commercial medical devices to further validate these formulations.

## Figures and Tables

**Table 1:** Scores considered for the evaluation of inflammation and lesion area.

Inflammation Evaluation		
Parameter	Phenotype	Score
Purulent Material	Not Present	0
	Present	1
Erythema	Not Present	0
	Mild	1
	Moderate	2
	Pronounced	3
	Severe	4
Erythema Width	<5 mm	0
	≥5 mm	1
Swelling	Not Present	0
	Mild	1
	Severe	2
**Area Evaluation**		
**Parameter**	**Phenotype**	**Score**
Wound	Dry	0
	Moist	1
Lesion Size	≤25 mm	0
	≤30 mm	1
	≤35 mm	2
	≥35 mm	3
Granulation Tissue	Covering Wound	0
	Moderate	1
	Not Present	2
Scab	Fallen off	0
	Moderate	1
	Mild	2
	Not Present	3
Necrosis	Not Present	0
	Mild	1
	Moderate	2
	Pronounced	3
	Severe	4

**Table 2:** Statistical differences in the corrected absorbance identified between different groups. Results significances are presented through the symbol (*), according to the *p*-value, with one, two or four symbols corresponding to 0.01 < *p* ≤ 0.05, 0.001 < *p* 0.01, and *p* ≤ 0.0001, respectively. (ns = no statistically significant differences).

	24 h				72 h				120 h				168 h			
	GG-HNY	GG-HA- HNY	DMEM 10%	DMSO 10%	GG-HNY	GG-HA-HNY	DMEM 10%	DMSO 10%	GG-HNY	GG-HA-HNY	DMEM 10%	DMSO 10%	GG-HNY	GG-HA-HNY	DMEM 10%	DMSO 10%
**GG-HNY**		*	****	**		*	****	****		ns	*	****		ns	ns	****
**GG-HA-HNY**			****	****			****	****			ns	****			ns	****
**DMEM 10%**				****				****				****				****

**Table 3:** Statistical differences in the corrected absorbance identified between different groups. Results significances are presented through the symbol (*), according to the *p*-value, with two or four symbols corresponding to 0.001 < *p* 0.01 and *p* ≤ 0.0001, respectively (ns = no statistically significant differences).

		24 h			72 h			120 h			168 h	
	Cream FB022	DMEM 10%	DMSO 10%	Cream FB022	DMEM 10%	DMSO 10%	Cream FB022	DMEM 10%	DMSO 10%	Cream FB022	DMEM 10%	DMSO 10%
**Cream FB002**		ns	ns		ns	****		****	****		****	****
**DMEM 10%**			**			****			****			****

## Data Availability

The original contributions presented in this study are included in the article/Supplementary Materials. Further inquiries can be directed to the corresponding author.

## Supplementary Materials

The following supporting information can be downloaded at https://www.mdpi.com/article/10.3390/biomedicines13020508/s1, Table S1—Corrected absorbance assessed by the PrestoBlue^TM^ viability test for L929 in direct contact between L929 cells, GG-HNY, and GG-HA-HNY, up to 168 h. Results presented as Mean ± SEM; Table S2—Percentage of cell viability inhibition after the direct contact of L929 cells with GG-HNY and GG-HA-HNY up to 168 h. Results are presented as Mean ± SEM; Table S3—Corrected absorbance assessed by the PrestoBlue^TM^ viability test for of L929 in indirect contact between L929 cells with cream FB002, up to 168 h. Results presented as Mean ± SEM; Table S4—Percentage of cell viability inhibition after the indirect contact of L929 cells with the cream up to 168 h. Results are presented as Mean ± SEM; Table S5—Values of the Wound Area Score for each therapeutic group over the study period. Results are presented as Mean ± SEM; Table S6—Inflammation score values for each therapeutic group over the study period. Results are presented as Mean ± SEM; Table S7—Values of the Wound Closure Rate (%) for each therapeutic group over the study period. Results are presented as Mean ± SEM; Table S8—Values of the wound area for each therapeutic group over the study period. Results are presented as Mean ± SEM; Table S9—Histopathological Classification of Lesions following ISO 10993-6:2016. The overall histological classification is presented as Mean ± SEM.
