# Supplementary material for: Honey, Gellan Gum, and Hyaluronic Acid as Therapeutic Approaches for Skin Regeneration
Source: Biomedicines. 2025 Feb 18;13(2):508. doi: 10.3390/biomedicines13020508 (PMC11853393; doi:10.3390/biomedicines13020508)
Supplement: Supplementary file 1 [file biomedicines-13-00508-s001.zip › biomedicines-3456768-supplementary.pdf]

Supplementary Material

Table S1 - Corrected absorbance assessed by the PrestoBlue™ viability test for of L929 in direct contact between L929 cells, GG-HNY and GG-HA-HNY, up to 168 hours. Results presented as Mean ± SEM.

	GG-HNY	GG-HA-HNY	DMEM 10%	DMSO 10%
**24h**	0.02108 ± 0.003	0.03158 ± 0.0005	0.05175 ± 0.002	0.003750 ± 0.002
**72h**	0.06008 ± 0.003	0.07558 ± 0.004	0.2059 ± 0.003	-0.01588 ± 0.001
**120h**	0.2740 ± 0.008	0.2900 ± 0.008	0.2958 ± 0.006	0.007833 ± 0.002
**168h**	0.2824 ± 0,009	0.2908 ± 0.002	0.296 ± 0.006	0.0078 ± 0.002

Table S2 - Percentage of cell viability inhibition after the direct contact of L929 cells with GG-HNY and GG-HA-HNY up to 168 hours. Results are presented as Mean ± SEM.

	GG-HNY	GG-HA-HNY	DMSO 10%
**24h**	59.26 ± 6.6	38.97 ± 0.9	92.75 ± 4.7
**72h**	70.82 ± 1.5	63.29 ± 2.0	107.6 ± 0.5
**120h**	7.35 ± 1.0	1.94 ± 2.5	97.35 ± 0.6
**168h**	4.5 ± 3.0	1.69 ± 0.6	97.4 ± 0.6

Table S3 - Corrected absorbance assessed by the PrestoBlueTM viability test for of L929 in indirect contact between L929 cells with cream FB002, up to 168 hours. Results presented as Mean ± SEM.

	Cream FB002	DMSO 10%	DMSO 10%
**24h**	0.003 ± 0.013	0.003 ± 0.013	-0.009 ± 0.002
**72h**	0.030 ± 0.000	0.030 ± 0.000	-0.031 ± 0.004
**120h**	0.0876 ± 0.0145	0.0876 ± 0.0145	-0.0664 ± 0.003
**168h**	0.0627 ± 0.0032	0.0627 ± 0.0032	-0.0757 ± 0.0020

Table S4 - Percentage of cell viability inhibition after the indirect contact of L929 cells with the cream up to 168 hours. Results are presented as Mean ± SEM.

	Cream FB002	DMSO 10%
**24h**	90.56 ± 44.73	130.00 ± 6.93
**72h**	46.53 ± 0.52	154.20 ± 8.23
**120h**	48.98 ± 8.23	138.70 ± 1.68
**168h**	80.29 ± 0.99	123.80 ± 0.62

Table S5 – Values of the Wound Area Score for each therapeutic group over the study period. Results are presented as Mean ± SEM.

Day	Sham	GG-HNY	GG-HA-HNY	Cream
0	7.00 ± 0.00	7.00 ± 0.00	7.00 ± 0.00	7.00 ± 0.00
4	6.00 ± 1.00	4.75 ± 1.09	4.50 ± 2.06	6.00 ± 1.58
8	5.25 ± 0.43	4.50 ± 0.50	4.75 ± 0.83	5.50 ± 1.12
12	3.75 ± 1.09	4.00 ± 0.71	3.75 ± 0.43	4.50 ± 0.50
16	2.00 ± 0.71	3.00 ± 1.22	3.00 ± 0.71	2.75 ± 0.83
20	2.50 ± 0.87	2.00 ± 0.71	2.25 ± 1.09	2.50 ± 0.87
24	0.50 ± 0.50	1.50 ± 1.50	0.75 ± 0.83	1.00 ± 1.73
28	0.50 ± 0.87	0.75 ± 0.83	0.25 ± 0.43	0.25 ± 0.43
30	0.00 ± 0.00	0.00 ± 0.00	0.00 ± 0.00	0.00 ± 0.00

Table S6 – Inflammation score values for each therapeutic group over the study period. Results are presented as Mean ± SEM.

Day	Sham	GG-HNY	GG-HA-HNY	Cream
0	0.00 ± 0.00	0.00 ± 0.00	0.00 ± 0.00	0.00 ± 0.00
4	3.25 ± 1.48	1.50 ± 0.50	2.00 ± 0.71	3.75 ± 2.28
8	3.75 ± 1.79	0.75 ± 0.43	1.00 ± 0.71	3.75 ± 1.30
12	1.50 ± 1.12	0.50 ± 0.50	0.50 ± 0.50	1.75 ± 0.83
16	0.25 ± 0.43	0.00 ± 0.00	0.25 ± 0.43	0.25 ± 0.43
20	0.25 ± 0.43	0.25 ± 0.43	0.50 ± 0.50	0.00 ± 0.00

24	0.00 ± 0.00	0.00 ± 0.00	0.00 ± 0.00	0.00 ± 0.00
28	0.00 ± 0.00	0.00 ± 0.00	0.00 ± 0.00	0.00 ± 0.00
30	0.00 ± 0.00	0.00 ± 0.00	0.00 ± 0.00	0.00 ± 0.00

Table S7 - Values of the Wound Closure Rate (%) for each therapeutic group over the study period. Results are presented as Mean ± SEM.

Day	Sham	GG-HNY	GG-HA-HNY	Cream
**0**	0.00 ± 0.00	0.00 ± 0.00	0.00 ± 0.00	0.00 ± 0.00
**4**	-57.73 ± 15.34	-69.69 ± 23.67	-77.54 ± 52.40	-52.92 ± 24.57
**8**	-37.17 ± 10.20	-40.21 ± 15.76	-60.43 ± 40.83	-35.18 ± 11.47
**12**	20.81 ± 22.12	-36.01 ± 35.36	-51.03 ± 38.75	-7.98 ± 36.27
**16**	43.48 ± 14.08	-8.24 ± 32.81	-5.63 ± 26.20	41.84 ± 25.58
**20**	61.81 ± 7.66	26.89 ± 41.04	45.96 ± 15.34	69.01 ± 4.68
**24**	75.09 ± 13.64	85.27 ± 5.24	81.54 ± 10.91	87.34 ± 7.44
**28**	77.79 ± 8.80	89.67 ± 2.26	81.46 ± 10.81	90.02 ± 5.09
**30**	93.40 ± 2.68	95.64 ± 2.00	94.88 ± 1.93	94.66 ± 4.11

Table S8 - Values of the wound area for each therapeutic group over the study period. Results are presented as Mean ± SEM.

**Day**	**Sham**	**GG-HNY**	**GG-HA-HNY**	**Cream**
**0**	6.00 ± 0.00	6.00 ± 0.00	6.00 ± 0.00	6.00 ± 0.00
**4**	9.46 ± 0.92	10.18 ± 1.42	10.65 ± 3.14	9.18 ± 1.47
**8**	8.23 ± 0.61	8.41 ± 0.95	9.63 ± 2.45	8.11 ± 0.69
**12**	4.75 ± 1.33	8.16 ± 2.12	9.06 ± 2.32	6.48 ± 2.18
**16**	3.39 ± 0.84	6.49 ± 1.97	6.34 ± 1.57	3.49 ± 1.53
**20**	2.29 ± 0.46	4.39 ± 2.46	3.24 ± 0.92	1.86 ± 0.28
**24**	1.41 ± 0.91	0.88 ± 0.31	1.11 ± 0.65	0.76 ± 0.45
**28**	1.33 ± 0.53	0.62 ± 0.14	1.11 ± 0.65	0.60 ± 0.31
**30**	0.40 ± 0.16	0.26 ± 0.12	0.31 ± 0.12	0.32 ± 0.25

Table S9 - Histopathological Classification of Lesions following ISO 10993-6:2016. The overall histological classification is presented as Mean ± SEM.

	Sham	GG-HNY	GG-HA-HNY	Cream
**30 days**	33.167 ± 3.380	27.500 ± 9.691	27.083 ± 5.351	30.000 ± 7.173
ISO SCORE	**0.00**	**-5.667**	**-6.083**	**-3.167**
